# Association between handgrip strength and cognition in a Chinese population with Alzheimer’s disease and mild cognitive impairment

**DOI:** 10.1186/s12877-021-02383-8

**Published:** 2021-08-11

**Authors:** Hang Su, Xiaokang Sun, Fang Li, Qihao Guo

**Affiliations:** 1 Department of Gerontology, Shanghai Jiao Tong University Affiliated Sixth People’s Hospital, 600 Yishan Road, 200233 Shanghai, China; 2 Department of Neurology, Fuxing Hospital, Capital Medical University, Beijing, China

**Keywords:** Mild cognitive impairment (MCI), Alzheimer's disease (AD), Handgrip strength

## Abstract

**Background:**

This study aimed to explore the level and changes in handgrip strength among preclinical Alzheimer’s disease (AD) and AD patients and to evaluate the association between handgrip strength and cognitive function.

**Methods:**

A total of 1431 participants from the memory clinic of Shanghai JiaoTong University Affiliated Sixth People’s Hospital and community were enrolled in the final analysis, including 596 AD, 288 mild cognitive impairment (MCI), and 547 normal individuals (NC). All participants received a comprehensive neuropsychological assessment. Mini-Mental State Examination (MMSE), Montreal Cognitive Assessment-Basic (MoCA-BC), and the Chinese version of Addenbrooke’s Cognitive Examination III (ACE-III-CV) were used as cognitive tests. The receiver operating characteristic curve (ROC) was plotted to assess the power of handgrip strength as a screening measure to discriminate AD and MCI.

**Results:**

The results showed that handgrip strength in the MCI group was significantly lower than that of NC group, and the AD group had a further decline (both *P < 0.01*). Multivariate logistic regression was performed with the handgrip strength quartiles, and the results showed that the ORs of AD for increasing levels of handgrip strength were 1.00, 0.58 (0.46–0.78), 0.51 (0.36–0.73), and 0.50 (0.35–0.68), showing a decreasing trend (*P*_*for trend*_ < *0.01*). The ROC curve demonstrated that the handgrip strength cutoff points for the identification of AD were 16.8 and 20.7 kg among the female participants above and under 70 yrs and 24.4 and 33.3 kg for the male participants above and under 70 yrs, respectively. Similarly, for the identification of MCI, cutoff points were 17.5 and 21.9 kg for females above 70 yrs and under 70 yrs, and 25.8 and 36.2 kg for males above 70 yrs and under 70 yrs, respectively.

**Conclusions:**

Our study provided the further knowledge on the relationship between noncognitive features and cognition in populations with differing cognitive status, revealed that the stronger handgrip strength was associated with better performances on cognitive function. It can be speculated that handgrip strength can help early recognition of Chinese AD patients.

## Background

Alzheimer’s disease (AD) is the most common type of dementia, with typical clinical symptoms appearing probably decades after progressive cognitive function loss with the initial phase of subtle changes [1]. Identifying predictive clinical signs of cognitive decline and dementia is imperative for the implementation of an adapted treatment [2]. Thus, preclinical AD research is of great significance to study the dynamics of AD pathogenesis and provide supporting evidence for early prevention strategies [3, 4]. Once cognitive impairment progresses to dementia, it can hardly be reversed. Mild cognitive impairment (MCI) is considered a transitional phase between normal cognitive aging and AD [5, 6]. The latest high-quality research suggested that amyloid accumulation, neuronal loss, and cognitive impairment may have progressively occurred at this stage [7, 8].

Previously, studies have shown that in addition to cognitive impairment, AD can also be accompanied by the performance of other systems of the organism, such as fatigue, slowed gait, and decreased muscle strength. These noncognitive features may become early predictors of cognitive impairment. Grip strength is one of the main indicators of body muscle strength and physical state, and it is also a relatively simple evaluation method [9]. Several studies have reported that poor handgrip strength is associated with a greater risk of cognitive impairment [9–13]. Furthermore, studies have suggested that higher handgrip strength at baseline is a protective factor in preventing the development of AD [14]. AD is preceded by a ‘silent’ clinical period that can last longer than a decade. Identifying such ‘soft’ physical signs associated with the progressive decline of cognitive function was significant for early intervention [9, 14]. The two main factors influencing handgrip strength are sex and age, where sex represents the largest proportion of the total variability [14]. However, few studies have explored the relationship between handgrip strength and cognition in preclinical AD participants, and the relationship is especially unclear among the Chinese population. Moreover, sex and age specific cut-off points of AD screening for handgrip strength have not been reported in previous studies. This study aimed to evaluate the association between handgrip strength and cognitive function and provide an analysis of AD screening efficiency for handgrip strength in a large population sample, and further investigate the optimal cutoff points according to different age and sex.

## Methods

### Participants

A total of 1431 participants were recruited from the memory clinic of Shanghai Jiao Tong University Affiliated Sixth People’s Hospital and the community in Shanghai. This study was approved by the Institutional Ethics Reviewing Board of Shanghai Jiao Tong University Affiliated Sixth People’s Hospital. Each subject had a uniform structured evaluation performed by a neurologist, which included a medical history inquiry and neurological examination. The study population was restricted to those without diseases that may have pathological effects on cognitive function. The inclusion criteria were normal vision and hearing to complete cognitive tests and no history of alcoholism, drug abuse, or head trauma. The exclusion criteria of this cohort included age ≤ 40 or above 90 years, low education level (≤ 5 years), presence of neurological or psychiatric antecedents, diseases involving the central nervous system, and severe diseases such as cancer, kidney disease, and chronic liver disease. Relevant laboratory tests were carried out to exclude metabolic disorders, nutritional deficiencies and infectious diseases that may adversely affect cognitive function, such as abnormalities in folic acid, vitamin B12, thyroid function, and rapid plasma regain or treponema pallidum particle agglutination. Cranial magnetic resonance (MR) imaging scanning was performed routinely to exclude any potential causes of cognitive decline, such as cerebral infarction, subdural hematomas, hydrocephalus, intracranial tumors and infections. All Subjects underwent comprehensive physical examination and positron emission tomography (PET) imaging.

### Neuropsychology

All participants received a comprehensive neuropsychological assessment, which was carried out by trained raters who were blind to the diagnosis. Six neuropsychological tests in three cognitive domains were examined: AVLT 30-minute delayed free recall auditory verbal learning test (AVLT-N5) and AVLT recognition (AVLT-N7) for memory domain [15]; animal fluency test (AFT, total score) and 30-item Boston naming test (BNT, total score) for language domain [16, 17]; shape trails test (STT), parts A and B (time to completion) for executive domain [18]. Mini-Mental State Examination (MMSE) [19–21], Montreal Cognitive Assessment-Basic (MoCA-BC) [22, 23], and the third version of Addenbrooke’s Cognitive Examination (Chinese version, ACE-III-CV) [24] were also tested as global cognition. The Chinese version of ACE-R was translated and culturally adapted within the Chinese population. Followed the guidelines introduced for the translation and cultural adaptation of ACE-III, we made some adaptions based on this version of ACE-R and formed the Chinese version of ACE-III. The Chinese version of ACE-III consists of five cognitive domains with a total score of 100: 18 points for attention and orientation, 26 points for memory, 14 points for verbal fluency, 26 points for language, and 16 points for visuospatial abilities. A higher score indicates better cognitive function [24]. Activities of Daily Living (ADL) and Functional Activities Questionnaire (FAQ) were used to assess functional capacity based on the reports of informants [25, 26]. Each neuropsychological test was standardized using published normative data and widely used in China with good reliability and validity.

### Measures

AD was diagnosed based on the recommendations from the National Institute on Aging-Alzheimer’s Association (NIA-AA) workgroups [27], which is ‘A/T/N’ system based on biomarkers investigated by PET and MR. MCI was based on Jak and Bondi’s criteria [5, 6, 28], and a diagnosis of MCI was given if the participant met one of the following criteria: (1) impaired scores (defined as > 1 standard deviation (SD) below the age-corrected normative mean) on two of the six neuropsychological indexes in the same cognitive domain (AVLT 30-minute delayed free recall and AVLT recognition for memory, AFT and BNT for language, STT-A and STT-B for executive function); (2) impaired scores (defined as > 1 SD below the age-corrected normative mean) in each of the three cognitive domains. Individuals who did not meet all of these criteria and had no cognitive impairment were identified as normal individuals (NCs).

### Collection of clinical information

Height and body weight were measured, and body mass index (BMI) was calculated as weight/height^2^ (kg/m^2^). Handgrip strength (kg) was estimated using a dynamometer (WCS-100, Nantong, China). Participants were asked to squeeze the dynamometer for a practice trial using submaximal effort to determine their understandings of the procedure and the grip size adjustments. They were randomly assigned to start the test with their dominant or nondominant hand. To complete the test, participants were asked to use one hand to squeeze the dynamometer as hard as possible and repeat using the other hand for a total of three alternating hands. Similar to previous studies using this measure, we extracted the maximum value achieved using either hand as the summary measure [29, 30].

### Statistical analysis

SPSS, version 23.0 (SPSS, Inc., Chicago, IL, USA) was used for statistical analysis. In this study, all continuous variables are presented as the mean ± standard deviation (SD), and categorical data are presented as numbers (percentages). Pair analyses were carried out using paired Student’s *t*-tests and Wilcoxon signed rank sum tests. Intergroup comparisons of skewed data were conducted using the Kruskal-Wallis test. Spearman correlation analysis and logistic regression analysis were conducted to identify independent factors of cognitive status. Multivariable-adjusted linear regression analysis were conducted to identify the associations between handgrip strength and MMSE scores. The receiver operating characteristic curve (ROC) was plotted to assess the power of handgrip strength as a screening measure to discriminate AD and MCI. A two-tailed *P* value of less than 0.05 was considered to be statistically significant.

## Results

### Baseline characteristics

Overall, 1431 participants were enrolled in the final analysis, with 48.8 % men and 51.2 % women, and the mean age was 69.8 ± 9.9 years. Of the 1,431 participants, 596 (41.6 %) had AD, 288 (20.2 %) had MCI, and 547 (38.2 %) were NC. We observed no statistically significant differences between males and females for most characteristics (Table 1), except for males being more likely to have higher height (*P < 0.01*), education years (*P < 0.01*), and BMI than females (*P = 0.034*). There were no statistically significant differences in terms of MMSE, MoCA-BC, or ACE-III-CV scores between females and males (all *P > 0.05*), while both ADL and FAQ performed better in males (both *P < 0.01*). Females had significantly lower handgrip strength (20.4 ± 5.9 vs. 31.0 ± 8.6 kg, *P < 0.01*) than males.

**Table 1:** Demographic and clinical characteristics of study participants

Variable	All participants (*n* = 1431)	Male (*n* = 698)	Female (*n* = 733)	*P Value*
Age (years)	69.9 ± 9.8	70.1 ± 10.0	69.8 ± 9.6	0.180
Education (years)	10.6 ± 4.6	11.7 ± 4.3	9.6 ± 4.6	< 0.001
Height(cm)	161.5 ± 13.7	168.4 ± 8.3	155.9 ± 14.6	< 0.001
BMI (kg/m^2^)	22.9 ± 3.3	23.2 ± 3.2	22.6 ± 3.3	0.034
Handgrip strength (kg)	24.5 ± 8.8	31.0 ± 8.6	20.4 ± 5.9	< 0.001
AD (n,%)	596 (41.2 %)	293 (42.7 %)	303 (41.0 %)	0.287
MCI (n,%)	288 (20.2 %)	145 (20.8 %)	143 (19.5 %)	0.401
MMSE	22.5 ± 7.1	22.7 ± 6.8	22.4 ± 7.3	0.884
MoCA-BC	18.7 ± 7.9	18.6 ± 8.0	18.9 ± 7.9	0.919
ACE-III-CV	62.1 ± 22.0	62.0 ± 22.4	62.3 ± 21.1	0.232
ADL	24.5 ± 9.2	23.9 ± 8.6	25.4 ± 10.2	<0.001
FAQ	5.1 ± 7.7	4.7 ± 7.4	5.8 ± 8.1	0.004

Data are presented as mean ± standard deviation or numbers (percentages)

*ADL* Activities of Daily Living, *AD* Alzheimer’s disease, *ACE-III-CV* Chinese version of Addenbrooke’s Cognitive Examination III, *BMI *body mass index, *FAQ* Functional Activities Questionnaire, *MCI* mild cognitive impairment, *MMSE* Mini-Mental State Examination, *MoCA-BC* Montreal Cognitive Assessment-Basic

### Comparison of handgrip strength levels among NC, MCI, and AD

**Fig. 1:**
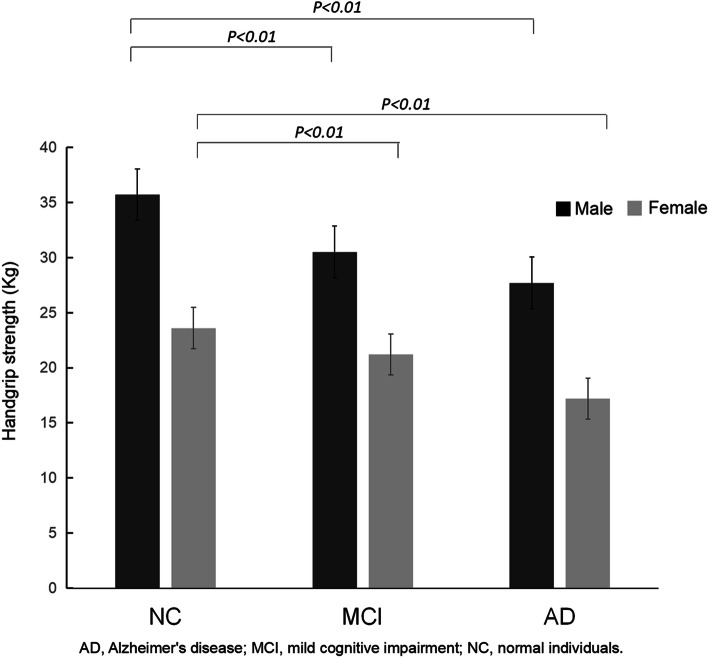
Characteristics of handgrip strength levels in participates with different cognitive function. Abbreviation: AD, Alzheimer's disease; MCI, mild cognitive impairment; NC, normal individuals

The handgrip strength among cognitive functions was compared in both the male and female groups. As shown in Fig. 1, the handgrip strength of the NC, MCI, and AD groups in males was 35.8 ± 7.3 kg, 30.4 ± 7.5 kg, and 27.7 ± 8.6 kg, respectively. The handgrip strengths of the NC, MCI, and AD groups in females were 23.6 ± 3.8 kg, 21.1 ± 5.6 kg, and 17.2 ± 5.7 kg, respectively. The results suggested that handgrip strength showed a decreasing trend with the decline of cognitive function in both the male and female groups (both *P*_*for trend*_*<0.01*). The level of handgrip strength in the MCI group was significantly lower than that in the NC group, and the AD group had a further decline (both *P < 0.01*).

### Associations between handgrip strength and cognitive function

**Fig. 2:**
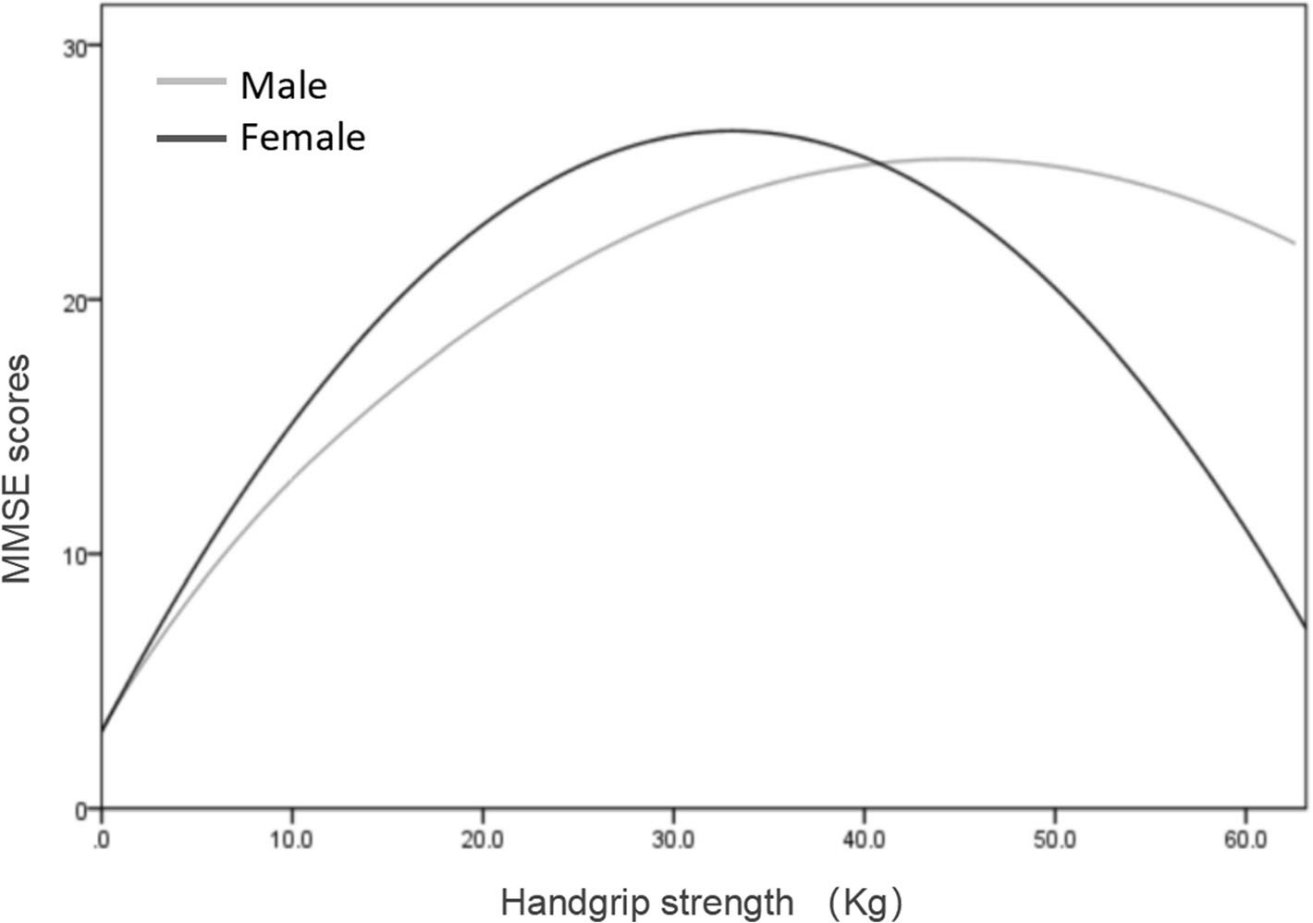
Sex-specific fitting curve of handgrip strength and MMSE scores. Abbreviation: MMSE, Mini-Mental State Examination

After dividing the population into sex subgroups of different sexes, spearman correlation analysis revealed that handgrip strength was negatively associated with age, FAQ, and ADL scores (*all P < 0.01*) and positively associated with BMI, MMSE, MoCA-BC, and ACE-III-CV scores (Table 2, *P < 0.01*). Multivariate logistic regression was performed with the handgrip strength quartiles, and the independent variables were different stages of cognition. After adjusting for age and BMI, the results showed that handgrip strength tended to decrease with the decline of cognition in subjects with AD and MCI (Table 3, both *P*_*for trend*_ < *0.01*). Multivariable-adjusted ORs of AD and MCI for increasing levels of handgrip strength were 1.00, 0.58 (0.46–0.78), 0.51 (0.36–0.73), and 0.50 (0.36–0.68) and 1.00, 0.36 (0.26–0.49), 0.29 (0.21–0.40), and 0.16 (0.10–0.21), respectively, showing a decreasing trend (both *P*_*for trend*_ < *0.01*). The fitting curve of handgrip strength and MMSE scores is shown in Fig. 2. Further multivariable-adjusted linear regression showed that each increase in kg of handgrip strength was associated with 0.348 (*95 % CI*: 0.214 to 0.357) and 0.450 *(95 % CI*: 0.422 to 0.528) higher MMSE scores in males and females, respectively (both *P* < *0.01)*.

**Table 2:** Spearman correlation of relationship between of handgrip strength and age, BMI, education years, and neuropsychology test scores

	Male		Famale	
	*r*	*P*	*r*	*P*
Age	−0.566	< 0.001	−0.503	< 0.001
BMI	0.492	< 0.001	0.512	< 0.001
Education years	0.128	0.416	0.188	0.302
MMSE	0.440	< 0.001	0.558	< 0.001
MoCA-BC	0.430	< 0.001	0.443	< 0.001
ACE-III-CV	0.418	< 0.001	0.436	< 0.001
FAQ	−0.481	< 0.001	−0.436	< 0.001
ADL	−0.502	< 0.001	−0.437	< 0.001

*ADL* Activities of Daily Living, *ACE-III-CV*,Chinese version of Addenbrooke’s Cognitive Examination III, *BMI* body mass index, *FAQ* Functional Activities Questionnaire, *MMSE* Mini-Mental State Examination, *MoCA-BC* Montreal Cognitive Assessment-Basic

**Table 3:** Odds ratios of participants with AD and MCI by quartiles of handgrip strength

	All participants		Male		Female	
	AD	MCI	AD	MCI	AD	MCI
Q4	1.00	1.00	1.00	1.00	1.00	1.00
Q3	0.58 (0.46–0.78)	0.36 (0.26–0.49)	0.54 (0.39–0.74)	0.31 (0.22–0.39)	0.63 (0.46–0.85)	0.40 (0.29–0.53)
Q2	0.51 (0.36–0.73)	0.29 (0.21–0.40)	0.48 (0.34–0.63)	0.26 (0.20–0.36)	0.54 (0.39–0.75)	0.33 (0.24–0.45)
Q1	0.50 (0.35–0.68)	0.16 (0.10–0.21)	0.45 (0.32–0.67)	0.14 (0.11–0.21)	0.53 (0.39–0.70)	0.17 (0.12–0.24)
*P*_*for trend*_	<0.001	<0.001	<0.001	<0.001	<0.001	<0.001

*AD* Alzheimer’s disease, *MCI* mild cognitive impairment

### Handgrip strength screening for the AD and MCI

**Fig. 3:**
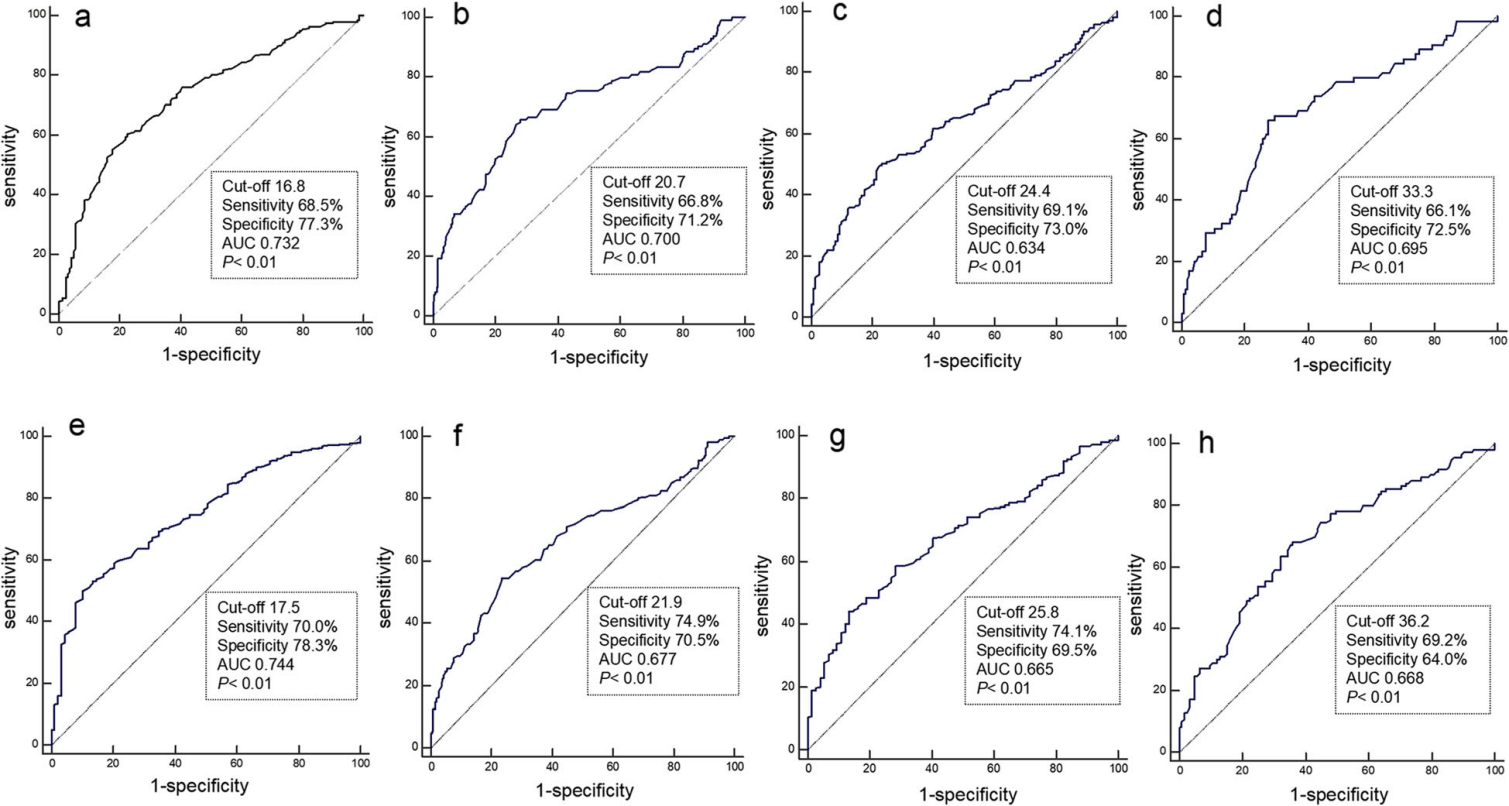
ROC curves for handgrip strength in the identification of AD (**a**. in the female above 70 yrs subgroup; **b**. in the female under 70 yrs subgroup; **c**. in the male above 70 yrs subgroup; **d**. in the male under 70 yrs subgroup) and MCI (**e**. in the female above 70 yrs subgroup; **f**. in the female under 70 yrs subgroup; **g**. in the male above 70 yrs subgroup; **h**. in the male under 70 yrs subgroup). Abbreviation: AD, Alzheimer's disease; MCI, mild cognitive impairment; ROC, receiver operating characteristic curve

Taking into account the influence of age and sex on handgrip strength, we performed ROC curve analysis separately in different sex and age subgroups. Figure 3a-d presents the ROC curves for the handgrip strength detection of AD based on recommendations from NIA-AA. The analysis demonstrated that the handgrip strength cutoff point for the identification of AD was 16.8 kg for females ≥ 70 yrs, with a sensitivity, specificity, and area under the curve (AUC) of 68.5 % (95 % confidence interval [*CI*]: 63.3–74.2 %), 77.3 % (*95 % CI*: 68.1–83.8 %), and 0.732 (*95 % CI*: 0.686–0.775), respectively. The cutoff point of AD was 20.7 kg in females < 70 years old, with a sensitivity, specificity, and AUC of 66.8 % (*CI*: 62.2–73.8 %), 71.2 % (*95 % CI*: 64.3–78.5 %), and 0.700 (*95 % CI*: 0.617–0.734), respectively. For males above and under 70, the cutoff points were 24.4 and 33.3 kg, with a sensitivity, specificity, and AUC of 69.1 % (*CI*: 65.6–74.2 %), 73.0 % (*95 % CI*: 67.1–79.4 %), and 0.634 (*95 % CI*: 0.602–0.673), respectively, and 66.1 % (*CI*: 53.4–77.8 %), 72.5 % (*95 % CI*: 65.0–78.9 %), and 0.695 (*95 % CI*: 0.632–0.754), respectively. Similarly, for the identification of MCI, cutoff points were 17.5 and 21.9 kg for female participants above 70 yrs and under 70 yrs (Fig. 3e-f) and 25.8 and 36.2 kg for male participants above 70 yrs and under 70 yrs (Fig. 3g-h), respectively.

## Discussion

This study was the first to analyze and compare the levels of handgrip strength in Chinese preclinical AD and AD participants and to explore the association between handgrip strength and cognitive function. The results showed that the level of grip strength in the MCI group was significantly lower than that in the NC group, and the AD group had a further decline. We found that stronger handgrip strength was associated with better cognitive performance, and each increase in kg of handgrip strength was associated with 0.348 (*95 % CI*: 0.214 to 0.357) and 0.450 *(95 % CI*: 0.422 to 0.528) higher MMSE scores in males and females, respectively. Finally, we proposed sex-specific and age-specific cutoff points for the initial identification of AD and MCI using grip strength, which provides evidence for the early detection of AD in the Chinese population.

Handgrip strength is a noninvasive measure of physical health that has been widely used in research and clinical settings. It can represent total body muscle strength and can also be an overall indicator of the integrity of the central nervous system [31]. Weak grip strength may indicate sarcopenia or central nervous system damage, which accelerates functional limitation. Prior studies have established an association between handgrip strength and cognition in aging cohort studies [32, 12], and impaired cognitive function and functional limitation present a mutually aggravating process. A 2-year cohort study of older Japanese adults found a significant correlation between lower MMSE scores and greater odds of functional decline [33]. A single-blind randomized controlled trial with 5 years of follow-up showed that cognitive training could slow functional decline in self-reported instrumental activities of daily life [34]. Therefore, handgrip strength has been suggested as a useful tool in geriatric practice in monitoring cognitive function decline [31]. Our findings showed that lower handgrip strength suggested a high risk of cognitive impairment, especially regarding handgrip strength levels in the preclinical AD population. Each increase in kg of handgrip strength was associated with 0.348 (*95 % CI*: 0.214 to 0.357) and 0.450 *(95 % CI*: 0.422 to 0.528) higher MMSE scores in the male and female groups, respectively, with a U-shaped association curve, which is similar to previous studies [35]. In addition, we proposed cutoff points for the initial identification of AD and MCI using grip strength among different age and sex subgroups, and the results indicated that it is reasonable to employ grip strength for AD simple screening.

Mechanistically, our findings are in accordance with the most notable hypotheses known as the ‘common cause hypothesis’, which demonstrates that cognition and muscle strength may share the same brain regions and networks [36]. This form of bounded rationality provides a reasonably straightforward way to implement the concept that simple motor tests or physical functions could be studied as biomarkers for identifying patients at a higher risk of cognitive impairment and dementia. However, there is still no direct imaging evidence to prove the rationality of this theory. Although it could be speculated from some studies that brain areas between motor coordination and cognitive function have an overlap [37], we would need a significantly intuitive research design to prove and refine this theory.

There are some limitations in this study. First, this was a single-center study, and data from multiple centers are needed to further confirm the findings. Second, a longitudinal study needs to be conducted to investigate the association between handgrip strength and cognitive function in these participants at follow-up. Further studies need to be performed with a larger volume of samples to investigate the difference among different cognitive domain impairments.

## Conclusions

This study showed that the level of grip strength in the MCI group was significantly lower than that in the NC group, and the AD group had a further decline. We confirmed that stronger handgrip strength was associated with better cognitive function and proposed that grip strength can identify early AD patients in the Chinese population. The current study brings further knowledge on the relationship between non-cognitive features and cognition in populations with differing cognitive status. In the future, it is necessary to conduct developed research on the association between frailty and cognition.

## Data Availability

The datasets generated and/or analyzed during the current study are not publicly available due the policy but are available from the corresponding author on reasonable request.

## Abbreviations

ADL: Activities of Daily Living
AD: Alzheimer’s disease
ACE-III: Chinese version of Addenbrooke’s Cognitive Examination III
BMI: Body mass index
FAQ: Functional Activities Questionnaire
MCI: Mild cognitive impairment
MMSE: Mini-Mental State Examination
MoCA-BC: Montreal Cognitive Assessment-Basic
ROC: Receiver operating characteristic curve
NC: Normal individuals
